# Oxidative stress-mediated genotoxic effect of zinc oxide nanoparticles on *Deinococcus radiodurans*

**DOI:** 10.1007/s13205-020-2054-4

**Published:** 2020-01-24

**Authors:** Ragini Singh, Shuang Cheng, Sanjay Singh

**Affiliations:** 1 School of Agriculture Science, Liaocheng University, Liaocheng, Shandong China; 2 Division of Biological and Life Sciences, Ahmedabad University, Central campus, Navrangpura, Ahmedabad, Gujarat 380009 India

**Keywords:** *Deinococcus radiodurans*, Zinc oxide nanoparticles, Oxidative stress, Free radicals, DNA damage

## Abstract

Extensive use of nanomaterials in consumer products has invoked the concerns about interactions of nanoparticles with living organisms (including microorganisms). Zinc oxide nanoparticles (ZnO NPs) are well known for their antibacterial effect due to reactive oxygen species (ROS) generation. Therefore, their release into the environment is expected to raise major concern towards ecotoxicity. In the present study, we have studied the toxic effect of ZnO NPs on *Deinococcus radiodurans*, which is well known to show extraordinary resistant from the damaging effects of radiation. Result showed that ZnO NPs are significantly internalized into the bacterial cells and induce concentration-dependent toxicity with membrane damage. Genotoxicity studies revealed that ZnO exposure induces significant DNA damage to bacterial cells. All the observations evidenced that ZnO NPs induce significant ROS generation, protein oxidation and DNA damage with concomitant thiol depletion. Further, gene expression analysis showed that several DNA repair genes and metabolic pathway-related genes are downregulated upon ZnO NP exposure, with simultaneous increase in the expression of DNA damage response genes. Thus, the present study on toxicity of ZnO NPs on a model organism, *D. radiodurans,* inflicts the possible mechanism behind ZnO NP-mediated toxic effects on various other microbial organisms.

## Introduction

Recently, nanoparticles (NPs) have gained significant attention in industrial applications due to their unique optical, electronic, mechanical, and magnetic properties. These properties mainly depend on the shape, size, and composition of NPs which further lead to the high surface-to-volume ratio and surface reactivity (Bradford et al. 2009). NPs such as zinc oxide (ZnO), titanium dioxide (TiO_2_), manganese oxide (MnO) and silver NPs (AgNPs) are used in various personal care and food products, sunscreens, plastic paints, gas sensors, textiles, solar cells, treatment of waste water and as an antibacterial agent (Oberdorster et al. 2005; Sheela et al. 2012; Srivastava et al. 2013; Memon et al. 2018; Memon et al. 2015; Y Liu et al. 2015). Despite their beneficial aspects, harmful effects of NPs over human health and environment remain one of the major bottlenecks towards implementation of their real potential. Release of NPs in air, water, and landfills has been reported to cause adverse effects on environmental and human health (Daughton and Ternes 1999; Memon et al. 2015). In context to environment, risk assessment of NPs must also be performed after their release into the environment i.e. mobility, reactivity and their effect on microorganisms (Simon-Deckers et al. 2009). The release of NPs in the environment is expected to cause adverse effects over the microbes and other organisms.

Zinc, due to its antibacterial property, can effectively be utilized in the commercial products like ointments, creams, lotions, and toothpastes. ZnO is generally considered as low toxic, but its nanoparticle form is highly reactive and responsive with high absorptivity (Almoudi et al. 2018). Due to the unique piezoelectric, magnetic and optical properties, ZnO NPs have received tremendous attention and applied in many aspects of food and agriculture system (Emami-Karvani and Chehrazi 2011; Newton and Warburton 2007). Antibacterial aspects of ZnO NPs are mainly due to the membrane disruption (Brayner et al. 2006) or induction of intracellular ROS in bacterial cells (Jones et al. 2008). Jiang et al. demonstrated that in the presence of radical scavenger, bactericidal effect of ZnO NPs can be suppressed, which gives direct evidence of reactive oxygen species (ROS)-mediated antibacterial effect of ZnO NPs (Jiang et al. 2016). Release of Zn^2+^ in bacterial culture medium is also proposed to be one of the major mechanisms of antibacterial activity of ZnO NPs. It has also been reported that upon activation by ultraviolet (UV) and visible lights, ZnO NPs generate various harmful radicals such as hydroxyl radical, superoxide radical and hydrogen peroxide (H_2_O_2_). Due to the negative charge hydroxyl radical and superoxides are unable to penetrate the bacterial membrane, whereas, H_2_O_2_ can cross the cell membrane efficiently (Padmavathy and Vijayaraghavan 2008). ZnO NPs are reported to possess bactericidal effect on *Campylobacter jejuni,* a food-borne pathogen, by disrupting the cellular membrane and inducing oxidative stress in bacterial cells (Xie et al. 2011).

Despite its extensive antibacterial activity, use of ZnO NPs is limited due to its toxicological impact on human body. Exposure of ZnO NPs was reported to induce significant cytotoxic effect and DNA damage in cells. Reports have also shown that ZnO NPs can be accumulated in mouse liver and induces cellular injury. ZnO toxicological data obtained so far are inconsistent and conflicting. No reports are available which can clearly suggest actual concentration of ZnO NPs which can be harmful to human body (Almoudi et al. 2018).

There have been several strategies to reduce the toxic effect of ZnO NPs but keeping the antimicrobial effect intact. In one of the cases, coating of ZnO NPs with biocompatible molecule, poly-(*N*-isopropylacrylamide), exhibited antimicrobial activity at lower concentration (1.33 mM) without any appreciable toxicity to mammalian cells. Additionally, chitosan-ZnO bands was successfully utilized for the wound treatment in mice, infected with *P. aeruginosa*, *S. intermedicus*, and *S. hyicus* (Siddiqi et al. 2018). ZnO NPs coated with various polymers such as poly (hydroxybutyrate-co-hydroxyvalerate) (PHBV), poly (lactic acid) (PLA) or poly(vinyl chloride) (PVC) are also reported to be utilized as antibacterial food packaging materials (Ogunsona et al. 2019). Although all the studies of NPs have focused towards their effect on normal bacterial species, however, the effect of NPs over a resistant strain of bacteria has not been explored in great detail. In this context, we have chosen a stress-resistant bacteria *Deinococcus radiodurans* and studied the toxicity mechanism of ZnO NPs. *Deinococcus radiodurans* possesses unique and extraordinary resistance against UV radiation, desiccation, gamma radiation and mitomycin C exposure, due to their accurate and strong DNA repair mechanism and protection of proteins from oxidative damage and manganese complex (Slade and Radman 2011) (Daly et al. 2010). Enzymatic and non-enzymatic small molecule-based antioxidants are also some of the major factors for their exceptional protection property against oxidative stress (Slade and Radman 2011; Anaganti et al. 2015). So far, no comprehensive studies are made to illustrate the impact of nanomaterials (NMs) on the *D. radiodurans*. Several reports demonstrated the oxidative stress-mediated toxicity of ZnO NPs over several gram-positive and gram-negative bacterial strains (Emami-Karvani and Chehrazi 2011). Therefore, it was of considerable importance to explore the cell viability and oxidative stress levels upon ZnO NPs exposure in bacterial strain capable of withstanding the effects of radiation and free radicals. The chosen experimental model, *D. radiodurans,* was an appropriate organism for such studies as it will help to discover the toxic potential of ZnO NPs as well as novel DNA repair pathways which can be activated in response to ZnO NP-mediated DNA damage.

## Materials and methods

### Preparation of nanoparticle suspension

ZnO NPs (< 100 nm) were obtained from Sigma Chemical Co. Ltd. (St. Louis, MO, USA) and the stock suspension was prepared by suspending 1.5 mg of ZnO NPs in 10 mL of 0.22 μm filtered DI water. Stock suspension was sonicated (Sonics Vibra cell; Sonics & Material, Newtown, CT, USA) at 30 W for 10 min (pulse of 2 min ‘on’ and 1 min ‘off’).

### Culture of *D. radiodurans*

Briefly, 5 mL of *D. radiodurans* R1 (strain BAA 816), culture was allowed to grow overnight in TGY media (0.1% glucose, 1% tryptone and 0.5% yeast extract) at 32 °C under shaking (150 rpm) condition (Anaganti et al. 2015). Bacterial growth was monitored by measuring the turbidity of culture at 600 nm using a spectrophotometer. Overnight grown culture was then re-inoculated into fresh TGY media (at A600 = 0.1) and allowed to grow up in log phase (A600 = 0.65 ± 0.05) for further experiment.

### Exposure of *D. radiodurans* to nanoparticles

*Deinococcus radiodurans* cells were grown up to a log phase, and pellet was obtained by centrifugation at 4000 rpm for 5 min and washed twice with PBS. Bacterial cells (*A*_600_ = 0.65 ± 0.05) were treated with different concentrations of ZnO NPs (1, 10, 20, 40, 80 μg/mL) for 3 h at 32 °C in PBS.

### ZnO NPs uptake study by flow cytometer

*Deinococcus radiodurans* cells (~ 0.6 × 10^8^ CFU/mL) were treated for 3 h with different concentrations (1–80 μg/mL) of ZnO NPs. 200 μL of bacterial cells were diluted in 0.8 mL of PBS and 10,000 cells were acquired by a flow cytometer (FACS Calibur, BD Biosciences, CA) in each of the control (without NP exposure) and treated sample. Side scattering (SSC) intensity was recorded in each case. Forward scatter (FSC) and SSC were set to logarithmic scale. NPs without bacterial cells were run in parallel for each sample to minimize any interference. Data were plotted as fold change in SSC intensity.

### Interaction analysis of ZnO NPs in *D. radiodurans* by scanning electron microscope

After 3 h exposure of *D. radiodurans* cells to 80 µg/mL ZnO NPs, treated and control *D. radiodurans* cells were washed 3 times with PBS. Cell pellet was fixed with 1 mL of 2.5% glutaraldehyde for 30 min at 4 °C. Cells were dehydrated in different ethanol concentrations (15, 30, 50, 70%), each for 20 min and at last dehydrated in 100% ethanol for 20 min (3 times). Dehydrated cells were completely dried by keeping overnight and then samples were analyzed using SEM (Jeol JSM 6010 LA).

### Distribution study of ZnO NPs in *D. radiodurans* by transmission electron microscope

ZnO NPs (80 µg/mL) treated and control *D. radiodurans* cells were washed thrice with PBS. Cells were then fixed with 1 mL of 2.5% glutaraldehyde at 4 °C for 30 min. Cells were pellet down and washed with 0.1 M sodium cachodylate buffer followed by fixation in 100 µL of 2% osmium tetraoxide for 2 h at 4 °C. Fixed pellet was washed with sodium cachodylate buffer followed by dehydration of cells through different grades of ethanol, i.e. 30–70%, each for 20 min and at last dehydrated in 100% ethanol 3 times for 20 min. Samples were then embedded in araldite resin and kept for 72 h at 60 °C and were sliced into ultrathin sections by using ultra microtome (Leica UC7). Grids were examined under a TEM (JEOL JEM1400 Plus) at an accelerating voltage of 100 kV.

### Antibacterial study

Viability of *D. radiodurans* cells was analyzed by colony-forming unit. Bacterial cells were exposed to varying ZnO NP concentration (1–80 µg/mL) in PBS for 3 h. Further, 50 µL of control and treated cells were spread with the help of glass rod (sterilized) on the TGY agar plate and plates were incubated at 32 °C for 48 h followed by manual colony counting.

### Cell viability study by MTT assay

MTT reduction by cells corresponds to its viability and was determined as described previously (Anaganti et al. 2015). After exposure, 100 µL of control and ZnO NPs (1–80 µg/mL) treated bacterial cells were transferred to 96-well plates and incubated with 10 μL of MTT dye (5 mg/mL in PBS) for 3 h at 37 °C. The so-produced formazan crystals were allowed to dissolve in 100 μL of DMSO and incubated for 30 min at 37 °C followed by absorbance measurement at 570 nm using a multiwell plate reader (Biotek, Synergy HT spectrophotometer). Viability was calculated by the formula given below:

$$\% {\text{ viable}}\;{\text{cells}} = \left( {{\text{OD}}_{570} \;{\text{of}}\;{\text{ NMs}}\;{\text{treated}}\;{\text{cells}}/{\text{OD}}_{570} \;{\text{of}}\;{\text{control}}\;{\text{cells}}} \right)\,{\times}\,100$$

### Live–dead discrimination assay

Live–dead discrimination in ZnO NP-treated and control bacterial cells was done by propidium iodide (PI) uptake assay in flow cytometer. Control and treated bacterial cells were centrifuged, washed with PBS and incubated with 100 µL of PI for 15 min at room temperature and volume was raised up to 500 µL by adding PBS followed by live and dead bacterial cells analysis by red fluorescence acquired by FL2 channel of flow cytometry.

### Study of alteration in membrane potential of *D. radiodurans* by ZnO NPs

Membrane potential of bacterial cells was measured by using JC-1 dye. Treated and control bacterial cells were centrifuged at 4000 rpm for 5 min, washed with PBS and incubated with 500 µL of JC-1 dye (10 μM in PBS) at 37 °C for 15 min (in dark). Fluorescence of JC-1 dye was acquired by flow cytometry at FL-1 and FL-2 channel for green and red fluorescence, respectively.

For fluorescence measurement, 100 µL of JC-1 dye incubated treated and control *D. radiodurans* cells were transferred into black bottom 96-well plate. Excitation and emission at 490 and 525 or 590 nm wavelengths, respectively, was recorded by using multiwell plate reader. Data were plotted as fold change.

### Determination of intracellular reactive oxygen species

Intracellular ROS generation in ZnO NP-treated *D. radiodurans* cells was measured by DCFDA dye. 100 µL of control and ZnO NPs treated bacterial cells were transferred to 96-well black bottom plate and allowed to incubate with 100 µL H_2_DCFDA dye (20 µM in PBS) at 37 °C for 30 min in dark. Fluorescence intensity was measured at an excitation and emission wavelength of 485 and 528 nm, respectively, using a multiwell plate reader.

### Thiol content measurement

Control and ZnO NP-treated bacterial cells were centrifuged for 5 min at 4000 rpm to pellet down the cells and washed with PBS. Next, cells were sonicated for 3 min (3 s ‘on’ and 3 s ‘off’) and centrifuged at 10,000×*g* for 15 min. 500 μL of supernatant was mixed with 2.5 mL of 0.01% 5,5′-dithiobis-(2-nitrobenzoic acid) (DTNB) and allowed to incubate for 15–20 min in dark. Thiol content was measured by recording absorbance at 412 nm by a multiwell plate reader. Bradford method was used to determine the protein concentration in bacterial cell extract using bovine serum albumin (BSA) as standard. Estimated thiol content was normalized to cellular protein level and expressed as fold change in thiol μmol/mg protein.

### Alkaline unwinding assay

Control and treated bacterial cells were pelleted down by centrifugation (4000 rpm, 5 min) and re-suspended in 500 µL of Tris (50 mM), EDTA (100 mM, pH 8.0), 10% SDS and 20 g/L of proteinase K. Cells were allowed to incubate at 55 °C for 5 h. Further, sample was suspended in an equal volume of chloroform/phenol/isoamyl alcohol (PCI) (24/25/1), followed by centrifugation at 13,000×*g* for 10 min at 4 °C. Obtained aqueous layer was transferred to other tube and treated with RNAase (10 mg/mL) at 37 °C for 30 min. DNA was then precipitated by adding absolute ethanol and incubated at − 20 °C followed by centrifugation for 15 min at 13,000×*g*. Pellet was allowed to be air-dried and dissolved in Tris (10 mM), EDTA (1 mM, pH 8.0).

For assay, the above isolated DNA has been distributed in 3 parts for fluorescence measurement in single-stranded DNA (ssDNA), alkaline unwound DNA (auDNA) and double-stranded DNA (dsDNA). For measurement of fluorescence from dsDNA, 100 µL of DNA sample was mixed with NaCl (100 µL, 25 mM), SDS (2 µL, 0.5%) followed by addition of potassium phosphate (pH 6.9, 0.2 M) and 3 µL of bisbenzamide (1 mg/mL). All the components were incubated for 15 min in dark. For fluorescence measurement of ssDNA, DNA sample was boiled at 80 °C for 30 min for complete unwinding of DNA and the rest of the procedure was same as followed for dsDNA. auDNA fluorescence was measured as follows: initially, DNA sample was mixed with alkaline buffer, i.e. 50 µL of NaOH (50 mM) and kept on ice in dark for 30 min. Further, 50 µL HCl (50 mM) and 2 µL of 0.5% SDS were added in the sample and the mixture was passed through 21-gauge needle many times. Potassium phosphate buffer was added in the sample followed by 3 µL of bisbenzamide (1 mg/mL) addition.

Fluorescence in each case was acquired at an *E*_x_/*E*_m_ = 360/450 nm for each sample. *F* value (ratio of dsDNA to total DNA) was determined by:

$${\text{F}} = \left( {{\text{auDNA}} - {\text{ssDNA}}} \right)/\left( {{\text{dsDNA}} - {\text{ssDNA}}} \right)$$

### DNA fragmentation study

DNA fragmentation was determined by staining the control and treated bacterial cell with nucleus staining dye, DAPI. Bacterial cells were exposed to ZnO NPs (80 µg/mL) for 3 h and then centrifuged for 5 min at 4000 rpm. Obtained pellet was re-suspended in PBS and was fixed on slides by air dry method and permeabilized with methanol for 10 min. Bacterial cells were then stained with DAPI (1 µg/mL in PBS) for 10 min in dark and the cells were imaged by fluorescence microscope (DM 2500, Leica, Wetzlar, Germany).

### Protein oxidation study

Protein oxidation analysis was performed by instructions provided in kit purchased from Merck-millipore (Oxyblot™ Protein oxidation detection kit, cat no. S7150). In brief, bacterial cells were exposed to 40 and 80 µg/mL ZnO NP concentrations for 3 h and pelleted by centrifugation for 5 min at 4000 rpm. Pellet was resuspended in PBS and sonicated for 2 min at 25% amplitude (2 s ‘on’ and 5 s ‘off’) for 2 min. Protein concentration was estimated by Bradford assay using BSA as standard. 6 µg of protein samples were denatured with 6% SDS and derivatized by the 1X DNPH (2,4-dinitrophenylhydrazine) solution provided in the kit and allowed to incubate at RT for 15 min. Neutralizing solution was added in each sample and samples were run on 12% polyacrylamide gel and transferred on PVDF membrane for 3 h at the voltage of 300 mV. Membrane was blocked with skimmed milk (5%) in TBST for 2 h and then incubated overnight with primary antibody (at 4 °C) provided in the kit. Membrane was washed with TBST (3 times) and incubated with secondary antibody for 2 h followed by washing with TBST. Blot was developed using chemiluminescence and analyzed through ImageQuantLAS 500 software.

### Real-time polymerase chain reaction

Bacterial cells were treated with 80 µg/mL of ZnO NPs for 3 h and total RNA was isolated from the control and treated cells by Trizol method. Cell pellet was re-suspended in 100 µL TE buffer containing lysozyme (6 mg/mL) and 500 µL Trizol reagent was added into above solution followed by addition of 100 µL of chilled chloroform. Mixture was centrifuged at 12,000*g* for 15 min at 4 °C and subsequent three layers were formed. Clear aqueous supernatant containing RNA was taken off and quantified at 260 nm using a UV–visible spectrophotometer.

A 0.5 µg of purified RNA was used as a template for the generation of cDNA using the cDNA synthesis kit (Thermofisher). Gene of interest was amplified using specific primer sets (provided in supplementary information, table ESI 1) with syber green dye. The PCR (20 μL) contains cDNA template (2 μL), SYBR green dye (10 µL) (Thermoscientific) and gene specific primers (1 µL each of forward and reverse primer) and experiment was performed using Quant qRT-PCR. The program detail was 95 °C, 10 min, followed by 95 °C, 15 s; 60 °C, 30 s; 72 °C, 30 s (40 cycles). A relative expression level for each gene was calculated using comparative *C*_T_ values. *C*_T_ values of specific genes were normalized with *C*_T_ value of *gapdh* gene whose intensity did not get altered in treated cells when compared with control cells.

## Results and discussion

Cytotoxic and genotoxic effects of ZnO NPs towards many bacterial (*E. coli, C. jejuni*) (Xie et al. 2011; Dutta et al. 2013) and mammalian cells (Gong et al. 2017) have been extensively investigated by several research groups. Zinc is considered as an important element for the growth and survival of bacterial cells, however, their concentration beyond critical limits can severely inhibit the activity of functional enzymes of bacteria such as NADH dehydrogenase, glutathione reductase, and peroxidase. These events lead to the oxidative stress in bacterial cells (Gajjar et al. 2009). Therefore, in this study, we have explored the toxicity induced by ZnO NPs in a stress-resistant species of bacteria, *D. radiodurans* cells.

### ZnO NPs are internalized in bacterial cells

**Fig. 1:**
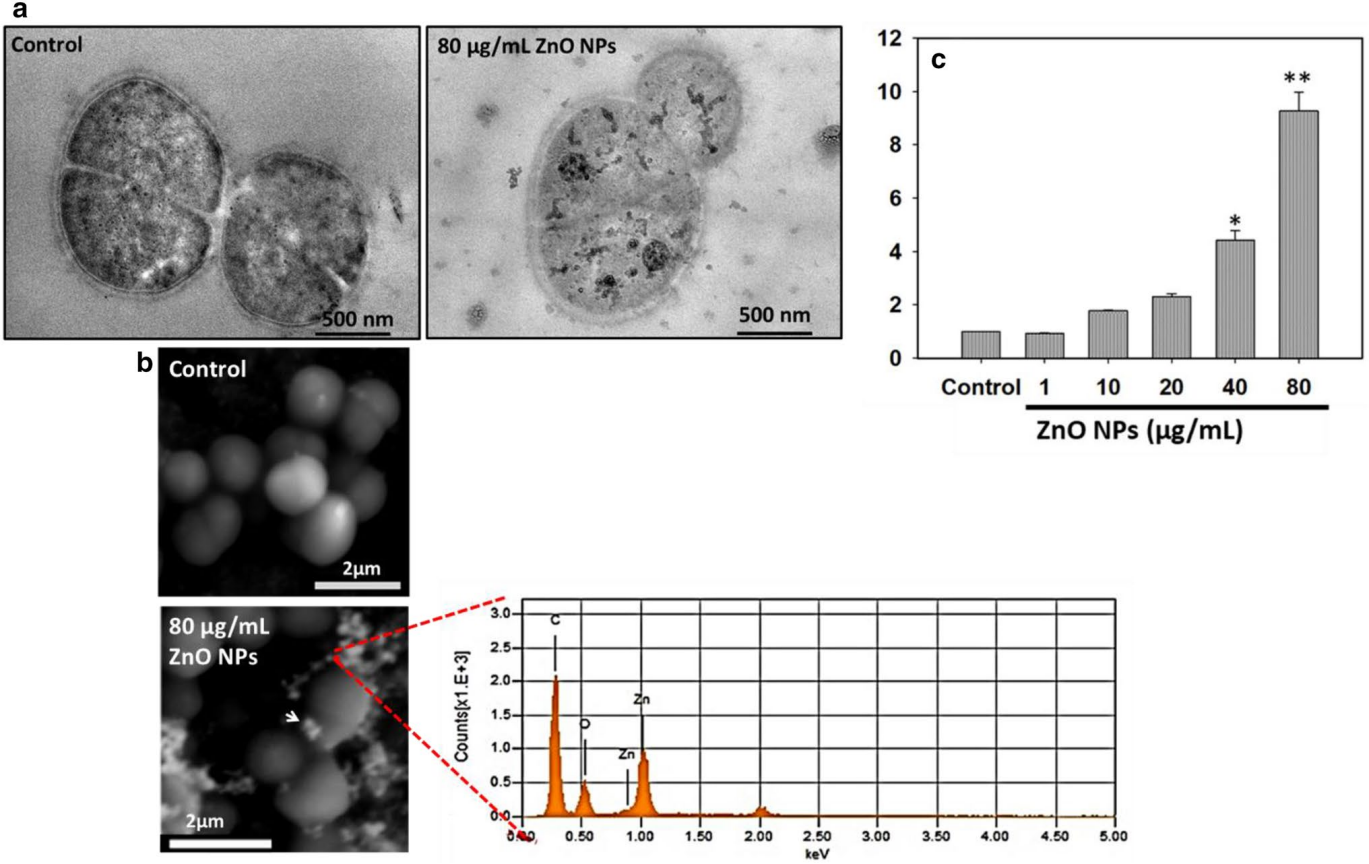
ZnO NPs were significantly internalized into *D. radiodurans* cells. **a** TEM images shows the internalization, **b** SEM images depicts the interaction of ZnO NPs on treated bacterial cells surface in comparison to control cells. **c** Flow cytometry graph shows the fold change in SSC and thus uptake intensity of ZnO NPs treated cells in comparison to control. Data expressed as standard error (SE) calculated from three (*n* = 3) independent experiments, *p < 0.05, **p < 0.01

It has been reported that the internalization of ZnO NPs in bacterial species such as *Salmonella typhimurium* and *E. coli* cells can be studied by transmission electron microscopy (TEM) (Brayner et al. 2006; Huang et al. 2008; Tam et al. 2008) and flow cytometry (Kumar et al. 2011b). Therefore, in the present study, we performed the uptake study of ZnO NPs in *D. radiodurans* by TEM (Fig. 1a, b) and flow cytometer (Fig. 1c). TEM image illustrates that ZnO NPs are significantly internalized and almost uniformly distributed into the bacterial cytoplasm (Fig. 1a). ZnO NP exposure induces membrane damage and also alters the morphology of bacterial cells with distorted natural tetrad form of bacteria. Interaction of ZnO NPs with bacterial cell surface was assessed by SEM imaging. SEM images along with elemental analysis by elemental dispersive X-ray analysis (EDAX) (Fig. 1b) suggest the presence of elemental zinc over bacterial cells, which further confirms their interaction with ZnO NPs. ZnO NPs are reported to increase the membrane permeability of bacterial cells, therefore, it is expected that the particles have internalized and accumulated within the cells (Brayner et al. 2006). Similar to our results, ZnO NPs are also reported to be internalized in *E. coli* cells followed by morphological changes of bacterial cells and few aggregates of particles at the cell surface (Kumar et al. 2011b). ZnO NPs are also reported to transform the spiral shape of *Campylobacter jejuni* into coccoid form (Xie et al. 2011) which was correlated with the nanoparticle-mediated membrane damage and oxidative stress.

Additionally, we also studied the uptake of ZnO NPs in bacterial cells by flow cytometer (Fig. 1c). *Deinococcus radiodurans* cells exposed to varying concentrations of ZnO NPs (1, 10, 20, 40 and 80 µg/mL) showed a significant increase in SSC intensity (1, 1.7, 2.3, 4.4, and 9 folds) than control untreated cells. This observation suggests that ZnO NPs are actively internalized inside the cells in a concentration-dependent manner and support our finding with TEM-based ZnO NP internalization (Fig. 1a). Internalization of NPs in bacterial cells is reported to follow the mechanisms such as specific uptake (by porins) through silCBA gene transportation system, non-specific membrane damage and non-specific diffusion but the method of quantitative estimation of NMs uptake in bacterial cells are still unidentified (Kumar et al. 2011a).

### ZnO NPs exposure reduces the viability of bacterial cells

**Fig. 2:**
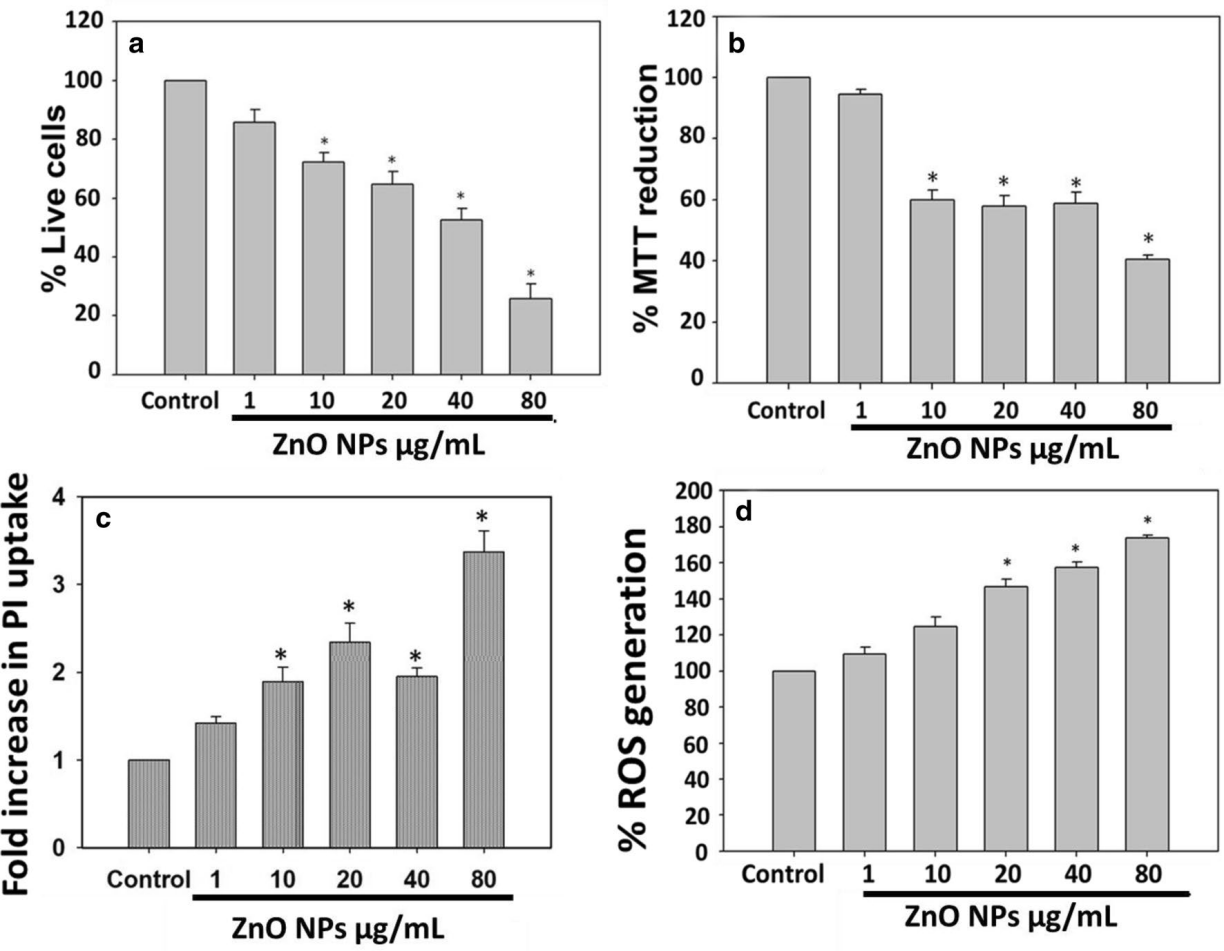
ZnO NPs are toxic to *D. radiodurans* cells. Cell viability was estimated by CFU, MTT and PI uptake assay whereas, ROS generation was estimated by DCFDA dye. **a**, **b** simultaneously represent the % decrease in cell viability of bacterial cells after 3 h exposure to ZnO NPs by CFU and MTT assay, respectively. **c** Represent the concentration dependent increase in PI uptake in bacterial cells. **d** Shows the increase in % intracellular ROS generation in bacterial cells by ZnO NPs exposure. Data expressed as standard error (SE) calculated from three (*n* = 3) independent experiments, *p < 0.05

Cell viability of *D. radiodurans* cells exposed to ZnO NPs was evaluated by three methods: conventional colony count method, MTT assay, and PI uptake assay. Result obtained by conventional colony count method revealed that exposure of ZnO NPs significantly reduced the viability of *D. radiodurans* cells in a concentration-dependent manner where 80 μg/mL ZnO NPs exposure reduced the cell viability to ~ 25% (Fig. 2a). MTT assay result also depicts that there is a significant reduction (~ 40%) in bacterial cell viability when exposed to 80 μg/mL concentrations of ZnO NPs, than untreated control cells (Fig. 2b). Additionally, PI uptake was also increased by ~ 3-fold in bacterial cells treated with 80 µg/mL ZnO NPs, than control cells (Fig. 2c). Cell viability results support our TEM observation (Fig. 1a) that ZnO NPs cause membrane damage in bacterial cells which further facilitates the entry of PI dye inside the cells. Various reports are available suggesting that electrostatic interaction of metal oxide NPs with bacterial cell surface induces membrane damage in cells (Fu et al. 2005; Stoimenov et al. 2002). Leaching of ions into culture medium is also reported to be one of the major causes of ZnO NP-mediated toxicity. Conventional plate count is a standard method, which only highlights the bacterial population capable of growing on agar plate and, therefore, used to quantify the biocidal efficiency of several antimicrobial agents. However, flow cytometry is a quantitative method, used to characterize the physiological state, such as permeabilized, damaged, and active bacterial population (Carre et al. 2018). Thus, in the present study, the toxicity of ZnO NPs was evaluated by conventional plate count method, MTT assay, and PI staining studied by flow cytometry. Result showed that toxicity observed in the plate count method was higher than observed in MTT and flow cytometry data, which could be due to the generation of “active but non-culturable” (ABNC) state of bacteria. In ABNC state, bacteria show significant physiological activities but unable to grow (Jung et al. 2008; Dolezalova and Lukes 2015), hence it is possible that after ZnO NPs treatment, *D. radiodurans* undergo ABNC state and thus fewer colonies were obtained (high toxicity) in conventional plate count method. However, due to its physiological active state, it shows more viability in MTT and PI uptake assays. This could be a possible reason to obtain different results in different viability methods.

### Deinococcus *radiodurans* exhibit intracellular ROS generation upon ZnO NP exposure

Interaction of NPs to bacterial cell surface leads to membrane damage and ROS production in cells, which could be one of the antibacterial mechanisms (Fu et al. 2005; Stoimenov et al. 2002). Thus, we subsequently evaluated the intracellular ROS generation in *D. radiodurans* cells in response to ZnO NP exposure (Fig. 2d). Bacterial cells exposed to ZnO NPs exhibited a significant concentration-dependent increase in intracellular ROS generation. Figure 2d shows that 20, 40 and 80 μg/mL of ZnO NP exposure causes 148, 157 and 189% increase in ROS generation, respectively, than control cells. Metal oxide NPs are well known to alter the microenvironment around the bacteria and thus induce ROS generation (Hajipour et al. 2012). It is well documented that smaller NPs can efficiently penetrate the bacterial cell membrane and inhibit the activity of respiratory enzymes (like NADH dehydrogenase II, a well-known site for ROS production) that leads to the ROS generation. Several different mechanisms are proposed for NP-mediated ROS generation (Song et al. 2009; Manke et al. 2013) such as, due to the unique surface characteristics of NPs. Interaction of NPs with cellular components or activation of NADPH-oxidase enzymes is some of the other proposed mechanisms (Manke et al. 2013). It has also been reported that the crystal defects produce high amount of electron–hole pairs at NP surface and contribute to the ROS generation. Electrons and holes react with oxygen and hydroxyl ions (in aqueous suspension) to produce highly reactive superoxide and hydroxyl radicals, respectively (Kumar et al. 2011b).

### ZnO NPs induce DNA damage in bacterial cells

**Fig. 3:**
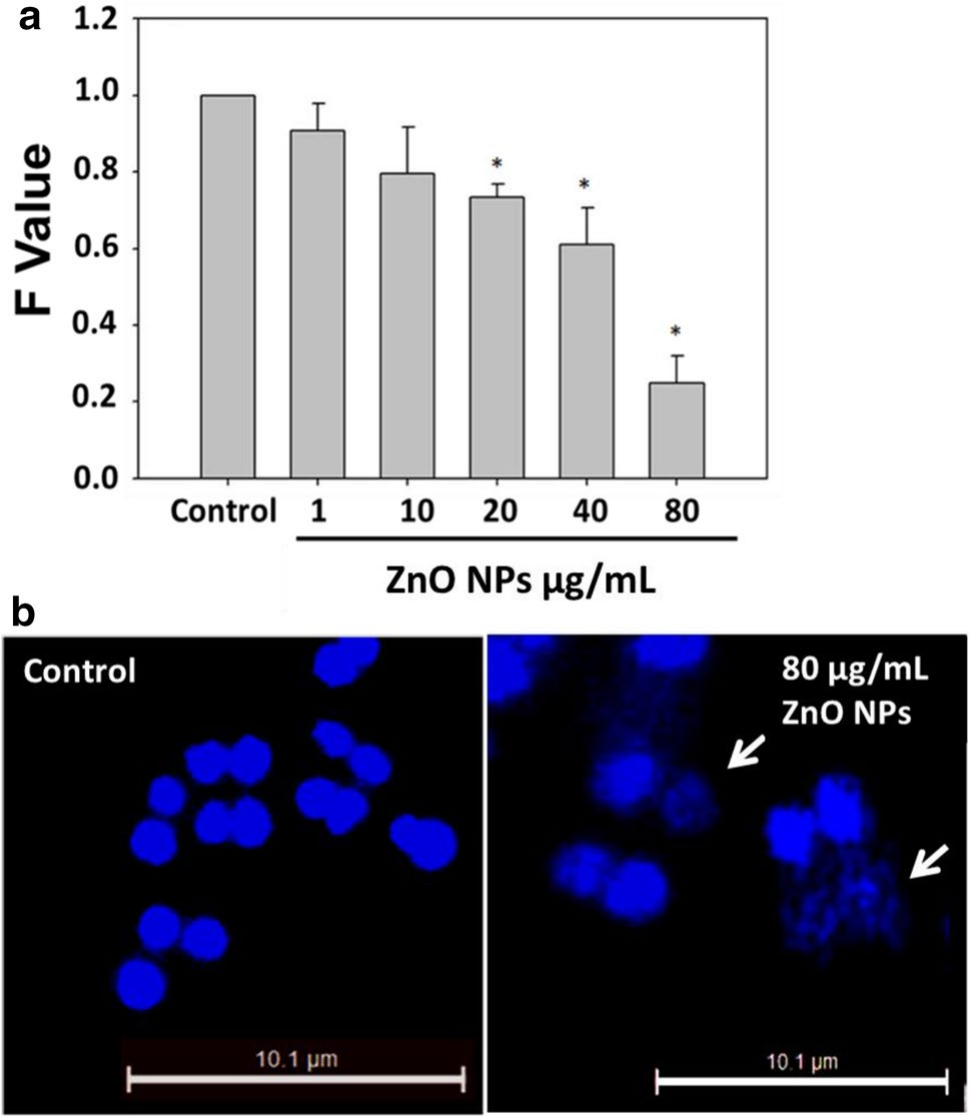
ZnO NPs are genotoxic to *D. radiodurans* cells **a** Alkaline unwinding assay, shows decrease in *F* value of treated cells which corresponds to increased DNA damage. **b** Fluorescent microscopy images of DAPI stained control and treated cells shows the significant amount of DNA fragmentation in ZnO NPs exposed cells (white arrow). Data expressed as standard error (SE) calculated from three (*n* = 3) independent experiments, *p < 0.05

NM-mediated ROS generation has been reported to be involved to cause genotoxicity along with cytotoxicity to cells (Singh et al. 2009). Metal oxide NPs can bind to the sulfur and phosphorous-containing membrane/cellular proteins or biomolecules such as DNA, which may lead to an adverse effect on the cells (Kumar et al. 2011b). Hence, we have also evaluated the DNA damaging potential of ZnO NPs in *D. radiodurans* by alkaline unwinding assay and DAPI staining. Results showed that ZnO NPs cause significant DNA damage in bacterial cells (Fig. 3). Unwinding assay revealed that “*F*” values decreases to 0.90, 0.79, 0.73, 0.60 and 0.25 fold at different ZnO NP concentrations, i.e. 1, 10, 20, 40 and 80 µg/mL, respectively, than untreated control cells (Fig. 3a). “*F*” value is inversely proportional to the DNA damage, therefore, decrease in *F* value after ZnO NP treatment corresponds to increased DNA damage. DAPI staining also showed similar trend of altered nuclear morphology (Fig. 3b, shown by white arrows) after ZnO NP exposure to bacterial cells. ROS can interact with DNA and produces chain breaks in nitrogenous bases and carbohydrates via nitration, methylation, deamination or oxidation reaction (Kumar et al. 2011b). Thus, correlation between cell viability, DNA damage and ROS generation proves that free radicals are the major causes of oxidative stress leading to cytotoxicity as well as genotoxicity in bacterial cells.

### ZnO NP exposure alters the membrane potential of bacterial cells

**Fig. 4:**
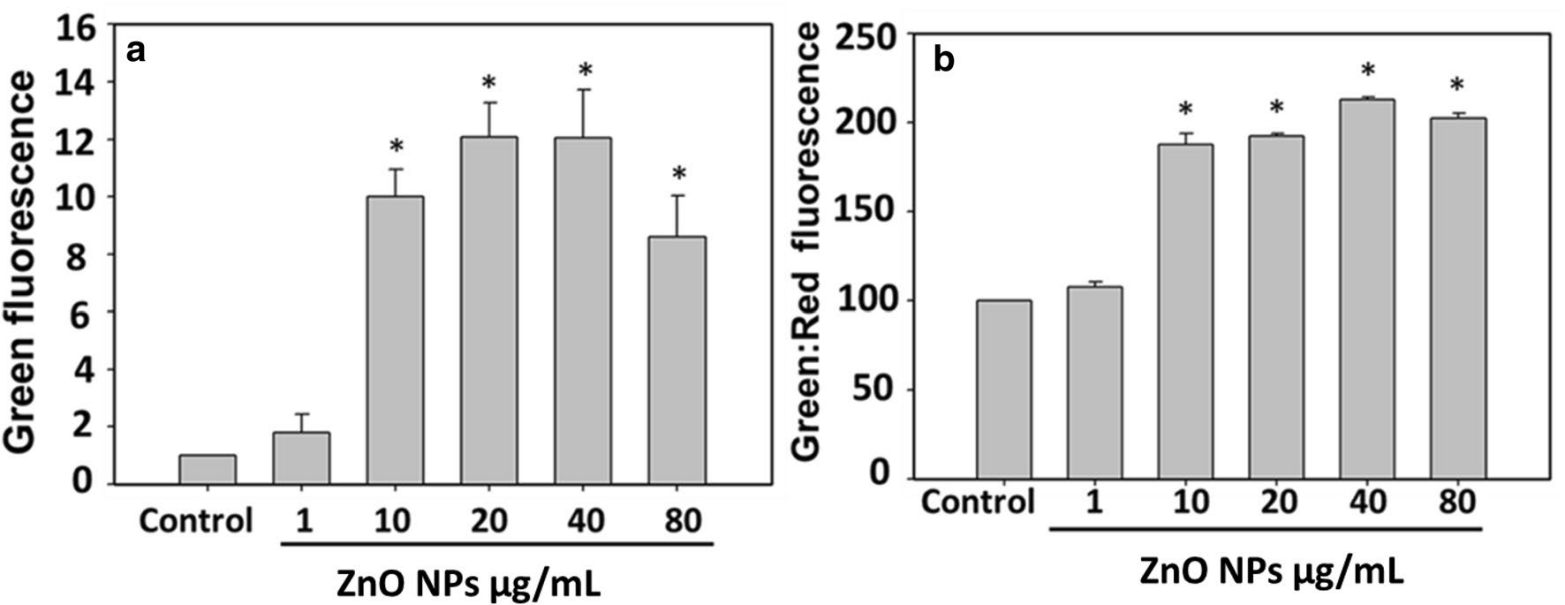
ZnO NPs cause decrease in membrane potential of bacterial cells. Fluorescent intensity of JC-1 dye was recorded by **a** Flow cytometry, **b** Plate reader in control and ZnO NPs treated cells. Data expressed as standard error (SE) calculated from three (*n* = 3) independent experiments, *p < 0.05

Membrane potential of ZnO NP exposed *D. radiodurans* was estimated by staining cells with JC-1 dye. Dye undergoes a change in colour from red to green due to the decrease in membrane potential. In healthy cells, JC-1 dye form aggregates leading to red fluorescence whereas, in cells undergoing apoptosis, dye displays green fluorescence and exists in monomeric form. Here green:red fluorescence ratio in treated and control bacterial cells was measured by two independent methods, i.e. flow cytometry and spectrophotometer. Flow cytometry data clearly shows that upon exposure to different concentrations of ZnO NPs (1–80 µg/mL), dose-dependent increase in green fluorescence (1.8, 9.9, 12.0, 12.0, 8.59 folds) was observed, which corresponds to membrane depolarization (Fig. 4a). Similar, result was also observed in the data obtained by recording the absorbance by spectrophotometer. As shown in Fig. 4b, green: red fluorescence was increased by 107, 187, 192, 212, 202% when *D. radiodurans* cells were exposed with 1, 10, 20, 40 and 80 µg/mL of ZnO NPs, respectively, for 3 h. Thus, these results clearly suggest that ZnO NP exposure reduces the membrane potential of *D. radiodurans* cells (Fig. 4).

### ZnO NPs challenge the antioxidant defense system in *Deinococcus ****radiodurans***

**Fig. 5:**
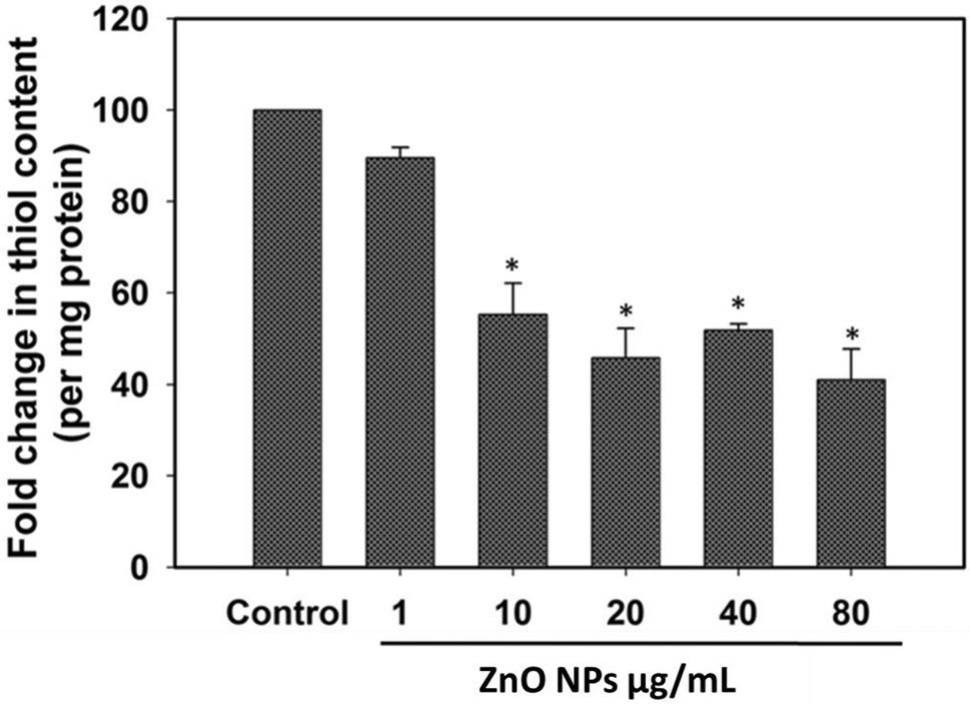
Antioxidant system of bacterial cells got challenged in presence of ZnO NPs. Thiol level in *D. radiodurans* cells was estimated after 3 h of ZnO NPs exposure. Data expressed as standard error (SE) calculated from three (*n* = 3) independent experiments, *p < 0.05

Thiol containing molecules have been proved to be an important constituent in genus *Deinococcus* which protects cells from oxidative stress. Intracellular thiol content is reported to acts as an antioxidant and it is suggested that the high thiol concentration imparts protection to *D. geothermalis* in response to oxidative stress (Kim et al. 2017). Additionally, molecules like bacillithiol, which is found in *Deinococcus* also act as an important antioxidant agent (Kim et al. 2017). Therefore, we also investigated the effect of ZnO NPs on thiol levels in *D. radiodurans*. Results illustrate that ZnO NP exposure induces concentration-dependent depletion of thiol levels in *D. radiodurans* cells (Fig. 5). Bacterial cells exhibit ~ 41.0% reduction in thiol content when exposed to various ZnO NP concentration. Thiol level depletion in *D. radiodurans* cells can be directly correlated with the increase in intracellular ROS generation due to the reduced cellular antioxidant defense system (Schins and Knaapen 2007). Similarly, tellurite exposure was also reported to induce decrease in the cellular thiol level of *D. radiodurans* leading to increased ROS generation and protein oxidation in bacterial cells (Anaganti et al. 2015).

### Protein carbonylation leads to oxidative stress in *Deinococcus ****radiodurans*** exposed to ZnO NPs

**Fig. 6:**
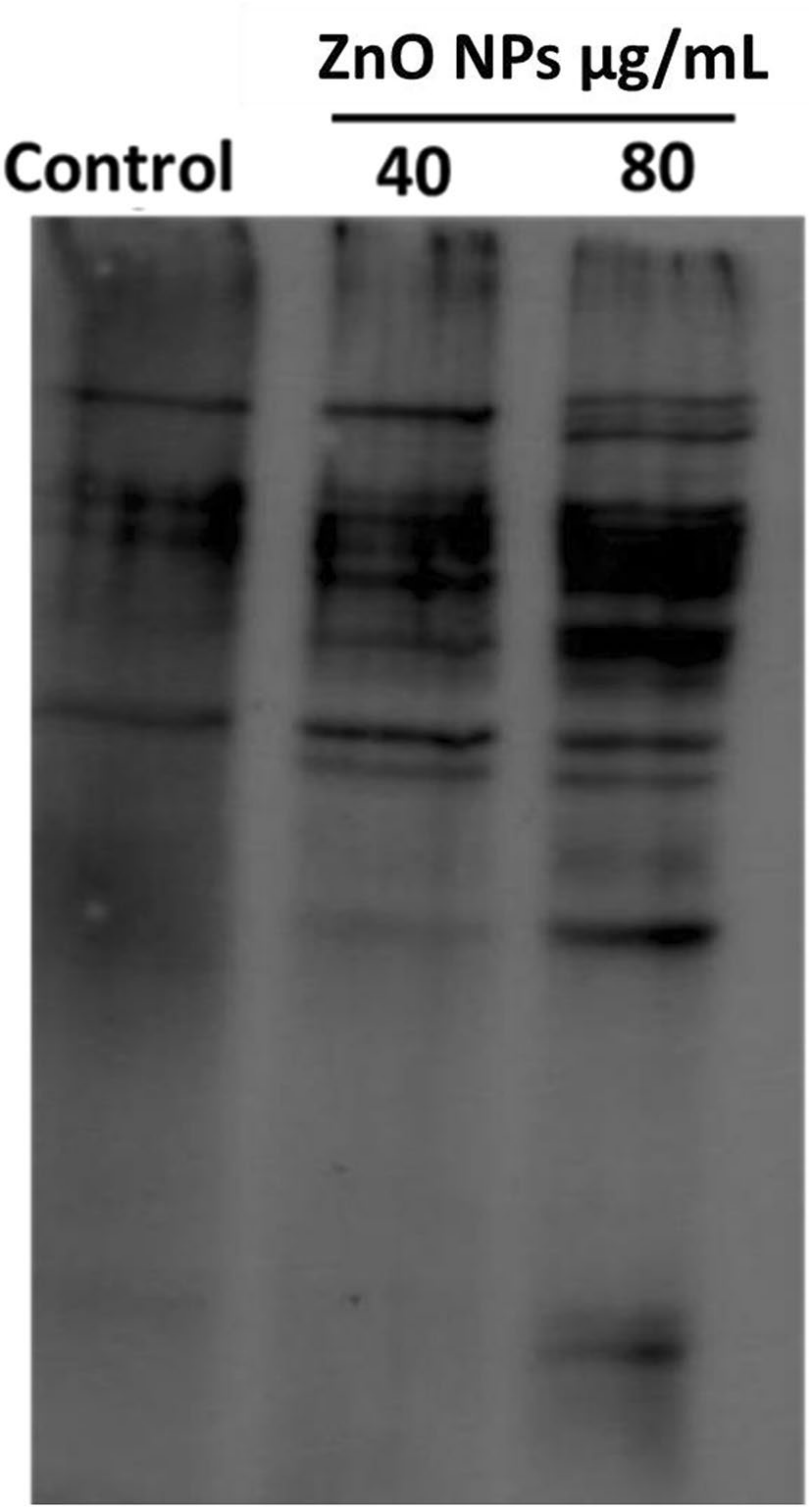
ZnO NPs induces damage to bacterial protein. Increase in protein carbonylation and thus oxidative stress level were recorded in ZnO NPs exposed cells (40 and 80 µg/mL) when compared to control cells

Free radicals are reported to induce structural modification in amino acids of cellular proteins (Lushchak 2001). ZnO NP-mediated oxidative stress in bacterial cells causes devastating effects on function and structure of cellular proteins (Kim et al. 2017). In our study, the damaging effects of ZnO NPs on *D. radiodurans* cellular proteins were estimated by analyzing the increase in protein carbonylation. Result showed that upon exposure to 40 and 80 µg/mL of ZnO NPs (for 3 h), protein carbonylation level was significantly increased in *D. radiodurans* (Fig. 6)*.* ROS generation distorts the protein structure and also leads to the oxidation of protein side chains that can be represented by a carbonyl marker (Krumova et al. 2009; Patra et al. 2015). These alterations in protein structure affect the normal functionalities of protein and disturb cellular redox balance, which could be one of the major reasons behind NP toxicity (Cabiscol Català et al. 2000).

### ZnO NP exposure affects the expression of several genes of metabolic pathways

To evaluate the role of various genes involved in metabolic, DNA damage repair and stress-related pathways, expression of these proteins was analyzed by following real-time PCR. Genes like Ddr A, Ddr B, SSB and DNA gyrase (involved in homologous recombination and multiple DNA repair pathways) are upregulated upon ZnO NP exposure. However, genes such as Mut S, Mut L (involved in mismatch repair pathways) are downregulated after 80 µg/mL ZnO NP exposure. Fold change in upregulation and downregulation of different analyzed genes is mentioned in Table 1.

**Table 1:** Expression pattern of genes involved in several DNA damage repair and metabolic pathways

Genes	Pathways involved	Expression
Rec A	Homologous recombination (HR)	+ 1.45
Ddr A		+ 10.96
Ppr A		+ 1.78
DNA helicase		− 4.24
IrrE		− 7.95
Mut L	Mismatch repair	− 2.16
Mut S		− 5.38
Ung	Base excision repair	− 6.61
Mut M		− 3.92
Uvr A	Nucleotide excision repair	− 1.12
Uvr B		− 1.46
SSB	Multiple pathways	+ 3.99
Ddr B		+ 24.6
DNA gyrase		+ 2.41
DNA polymerase		− 7.2
DNA ligase		− 5.47
Ddr C		− 3.27
Ddr D		+ 2.85
RNA ligase		+ 2.85
Dna K	Stress related pathways	+ 2.07
Thioredoxin reductase		+ 1.36
Catalase		− 1.07
Heat shock protein (HSP)		− 2.64
GTPase		− 1.46
SOD		+ 1.15
Y fit		− 5.06
Com A		− 5.44
DR1314		− 2.6
Aconitase	Metabolic pathways	− 2.6
Succinate dehydrogenase		− 5.26
Oxoglutarate dehydrogenase		− 2.33
Glucose kinase		− 3.9
Phospho fructo kinase		− 4.39
Pyruvate kinase		− 2.13
ATP synthatase A	ATP metabolism	− 3.2
ATP synthatase B		− 2.7
ATP synthatase C		− 3.54
Trans methylase	Random pathways	− 2.2
Etf B		+ 2.57
Lon 2		− 2.01

Following exposure to ZnO NPs, level of genes related to DNA repair pathways like mismatch repair (Mut S and Mut L), base excision repair (Ung and Mut M) and genes involved in DNA synthesis (DNA polymerase and DNA ligase) (Slade and Radman 2011) were found to be downregulated in ZnO NP-treated cells. The obtained results suggest that the role of DNA repair enzymes is challenged in ZnO NP-treated cells which could be one of the mechanisms behind ZnO NP-mediated toxicity. In contrast to the above results, upon ZnO NP exposure some DNA damage response genes like Ddr A, Ddr B, Ddr D are highly upregulated (up to 10- to 20-folds), along with SSB and gyrase A genes. Ddr A (protect 3′ end of DNA from degradation) (Harris et al. 2004) and Ddr B (strand annealing properties) (Norais et al. 2009) proteins are important for Rec-A independent single-strand annealing (SSA) (Slade and Radman 2011). Ddr A is known to preserve the genomic integrity of *D. radiodurans* by protecting the DNA fragment generated by nuclease activity, importantly in stress condition when DNA repair processes are inhibited (Harris et al. 2004). SSB protein is known to protect the ssDNA from nucleolytic degradation and help in Rec A filament formation. DDr B is alternative unique SSB protein found in *D. radiodurans* and proposed to display, single-strand annealing properties even in the presence of SSB and its depletion sensitizes the cells towards ionizing radiation (Slade and Radman 2011). Upregulations of Ddr A, Ddr B, SSB and gyrase A in ZnO NP-treated cells revealed that a unique DNA repair system of *D. radiodurans* attempts to provide resistance towards ZnO NP-induced DNA damage in bacterial cells. Genes related to nucleotide excision repair pathway (Uvr A and Uvr B) are important in the removal of mutation caused due to DNA-damaging effect of ZnO NPs (Luan et al. 2014). As shown in Table 1, Uvr A and Uvr B gene expression remain unaltered in ZnO NP-treated cells, which may be due to the non-involvement of this pathway in repair mechanism.

Several enzymes like aconitase, succinate dehydrogenase, glucose kinase, phosphofructo kinase, pyruvate kinase, and oxoglutarate dehydrogenase are significantly involved in the key metabolic pathways like glycolysis and TCA cycle. Expression of the genes encoding these enzymes is found to be significantly decreased upon NP exposure. Decreased level of these enzymes can severely affect the regeneration of molecular energy [NAD(P)H and ATP] in ZnO NP-exposed cells (Anaganti et al. 2015). Moreover, V-type ATP synthetase subunit A, B, C genes which are involved in the process of ATP synthesis from ADP were also analyzed in ZnO-exposed bacterial cells. Result showed that the expression of these genes is also downregulated in ZnO NP-treated cells, which causes ATP depletion in cells and can also be considered as one of the major reasons of ZnO NP toxicity in bacterial cells. Under stress condition, *D. radiodurans* cells suppress the energy generation and also limit the metabolic demands (Luan et al. 2014). Expression of antioxidant pathway-related genes like thioredoxin reductase (TrxR), catalase and superoxide dismutase (SOD) was not affected by NP treatment. Dnak is chaperone and involved in renaturation and functionality of oxidative damaged and denatured proteins are also upregulated in response to ZnO NP exposure suggesting their protective action against oxidative stress-mediated protein damage (Anaganti et al. 2015). Hence, it can be assumed that ZnO NP exposure leads to protein damage in bacterial cells, which is in support of data showing protein carbonylation as indication of oxidative damage. Further, Dr1314 (heat shock protection) (Liedert et al. 2012) is other stress-related protein which is found to be downregulated upon ZnO NP exposure. Lon2 is involved in the removal of misfolded proteins (Slade and Radman 2011) and also found to be downregulated after ZnO NP exposure. Thus, downregulation of key proteins which plays crucial role in stress protection can be considered as one of the major causes of oxidative stress in ZnO NP exposed *D. radiodurans*.

Thus, it can be concluded that if ZnO NP proved toxic to stress resistant bacteria it can induce severe toxicity to various other prokaryotes as well as eukaryotes. In this context, several reports have evidenced about the accumulation and toxicity of ZnO NPs in soil microorganisms, earthworms and various plants (Romero-Freire et al. 2017; Garcia-Gomez et al. 2015). Hence, there is an urgent requirement to investigate the safety of ZnO NPs in the various organisms including stress-resistant species.

## Conclusion

ZnO NPs display oxidative stress-mediated genotoxic and cytotoxic effects in *D. radiodurans*. NPs are rapidly internalized in bacterial cells and cause membrane damage with morphological alterations. Moreover, ZnO NPs significantly reduce the thiol levels with concomitant increase in free radical generation leading to DNA damage, protein oxidation and cellular membrane disruption. ZnO NPs also cause impairment of several genes involved in DNA repair pathways (Mut S, Mut M), metabolic pathways (aconitase, succinate dehydrogenase) and ATP synthesis (ATP synthase) of the bacteria. These events could also be considered as major causes of toxicity. However, upregulation of expression of several unique DNA damage response genes (ddr A, ddr B, ddr D) attempts to provide resistance against the ZnO NP-mediated DNA damage. Overall, the present study suggests that higher concentrations of ZnO NP exposure induces toxicity to *D. radiodurans*, however, the lower concentrations could be well tolerated by this organism. Therefore, in-depth investigation of mechanism of protection by *D. radiodurans* could pave a path to avoid the obvious toxicity of ZnO NPs to other economically important microbes.
